# Oral Health Status in Patients with Amyotrophic Lateral Sclerosis: A Scoping Review

**DOI:** 10.3390/dj13100455

**Published:** 2025-10-03

**Authors:** Leopoldo Mauriello, Alessandro Cuozzo, Vitolante Pezzella, Gaetano Isola, Gianrico Spagnuolo, Vincenzo Iorio-Siciliano, Luca Ramaglia, Andrea Blasi

**Affiliations:** 1 School of Dental Medicine, University of Naples Federico II, Via S. Pansini 5, 80131 Naples, Italy; leopmau96@gmail.com (L.M.); alessandro.cuozzo@unina.it (A.C.); gspagnuo@unina.it (G.S.); vincenzo.ioriosiciliano2@unina.it (V.I.-S.); luca.ramaglia@unina.it (L.R.); andrea.blasi@unina.it (A.B.); 2 Unit of Periodontology, Department of General Surgery and Medical-Surgical Specialties, University of Catania, 95124 Catania, Italy; gaetano.isola@unict.it; 3 Therapeutic Dentistry Department, Institute for Dentistry, Sechenov University, Moscow 119991, Russia

**Keywords:** amyotrophic lateral sclerosis, oral health, bulbar dysfunction, periodontal disease, multidisciplinary care

## Abstract

**Background**: Amyotrophic lateral sclerosis (ALS) is a neurodegenerative syndrome which often leads to progressive muscular dysfunction and therefore oral health deterioration. The aim of this scoping review is to evaluate oral health status in ALS patients focusing on the importance of dental care in improving patient’s quality of life. **Methods**: A comprehensive literature search was conducted on PubMed, Scopus, Web of Science, and Embase databases until June 2025 using a combination of keywords and MeSH terms related to ALS and oral health. Studies were screened and selected based on inclusion and exclusion criteria, focusing on human clinical data reporting oral health outcomes in ALS. **Results**: Eight studies met the inclusion criteria. The findings showed a high prevalence of oral complications in bulbar-onset ALS patients. Common issues included reduced tongue mobility, poor oral hygiene, sialorrhea, and decreased masticatory function were evaluated. **Conclusions**: Oral health impairment in ALS patients frequently contributes to systemic risks and reduced quality of life. A dental expert may play an important role in multidisciplinary care teams in terms of early diagnosis and conservative treatment of oral diseases ranging from periodontal disease to temporomandibular disorders (TMD). Personalized oral hygiene strategies and adjunctive therapies may serve as key elements in maintaining overall health and patient comfort in ALS. Therefore, the objective of the following review was to evaluate oral health complication in patients with ALS, highlighting the impact of oral care on patients’ quality of life.

## 1. Introduction

Amyotrophic lateral sclerosis (ALS) is a neurodegenerative disease which affects both upper and lower motoneurons [1]. Although, recently, ALS seems to be considered a syndrome rather than a disease due to the different cellular mechanism failure involved, suggesting an interaction between environmental factors and genetic predisposition for disease’s onset [1]. Oral health has gained increasing importance in medically vulnerable groups. Particularly, during the Coronavirus Disease 2019 (COVID-19) pandemic, when the interruption of oral care was noted from an increase in emergency visits with a drastic reduction in preventive care [2]. The same consideration can be made for patients with ALS, since physical disability and systemic impairment often prevent them from maintaining proper oral care and periodical dentist visits. Teledentistry may be used as a complementary tool to manage dental visits in compromised ALS patients (e.g., with muscle impairments). Therefore, integrating some sort of remote support could at least help to reduce complications such as periodontitis, dental caries and gingivitis. Finally, oral health plays a crucial role in modulating systemic diseases and inflammation, suggesting that a multidisciplinary approach in the treatments of these conditions may be useful for patients’ well-being [3].

The first signs and symptoms are weakness, progressive muscle atrophy, fatigue, difficulties in swallowing and at last respiratory distress [4]. The disease is rare; however, in Europe, an incidence of 2.08/100,000 was recorded, corresponding to approximately 15.000 cases [4]. Oral health was defined in 2016 by the World Dental Federation as “multi-faceted and includes the ability to speak, smile, smell, taste, touch, chew, swallow and convey a range of emotions through facial expressions with confidence and without pain, discomfort and disease of the craniofacial complex.” [5]. Periodontitis records a high prevalence and it is widespread all around the world, in fact, according to Trindade et al., the prevalence of periodontitis in the adult population is 61.6%, with a severe form recorded in approximately a quarter of the evaluated population [6]. Since ALS patients are often affected by several oral symptoms such as muscle dysfunction, including orofacial, lingual, and pharyngeal muscles weakness, their ability to perform correct oral hygiene maneuvers is rather low, increasing patients’ risk of developing tooth decay and periodontitis [7]. On the other side, dental caries, especially in populations with limited self-care ability, represents a critical dimension. Selwitz et al. recorded that dental caries also remains one of the most prevalent chronic diseases globally, caused by several factors such as dietary sugars, acidogenic bacterial biofilms, reduced salivary flow, and poor oral hygiene [8]. As a consequence, caries risk is higher in individuals with inadequate salivary flow and neuromuscular impairments conditions frequently observed in ALS [4]. Furthermore, periodontitis in ALS should not be underestimated. In fact, periodontal pathogenic bacterial products are able to increase host immune and inflammatory response leading to a well-known association between periodontitis and both cardiovascular and neurological diseases (e.g., Alzheimer’s disease) [7,9,10]. These factors may predispose ALS patients to systemic complications such as aspiration pneumonia, which remains one of the causes of mortality in this population. Therefore, the main aim of this review is to evaluate the oral health status of ALS patients in order to understand if major attention should be paid to oral diagnosis and treatment of these patients to improve their quality of life.

## 2. Materials and Methods

A protocol was developed in adherence to the PRISMA-ScR Checklist (Preferred Reporting Items for Systematic Reviews and Meta-Analyses—extension for Scoping Reviews) [11].

A hand and electronic search were conducted on the following databases: PubMed, Scopus, Web of Science and Embase. The string search was designed in order to identify clinical studies that focused on oral health status of patients diagnosed with Amyotrophic Lateral Sclerosis (ALS). Searches were conducted up to 15 June 2025, without data restrictions. The search strings used are reported in Appendix A.

### 2.1. Eligibility Criteria

The following inclusion and exclusion criteria were defined for the literature search:

Inclusion Criteria

- Studies where data on oral health status, oral hygiene, or dental conditions in patients with a confirmed diagnosis of ALS were reported;
- Case reports, clinical studies, clinical trials, controlled clinical trials and randomized controlled trials;
- Articles published in English language;
- Human studies.

Exclusion Criteria

- Reviews, meta-analyses, letters to the editor, conference and abstracts without full-text availability;
- Animal or in vitro studies;
- Studies where ALS diagnosis was not clearly defined or where data on oral health status as a primary or secondary outcome were missing;
- Duplicates or studies where full text was unavailable.

### 2.2. Study Selection and Data Extraction

**Figure 1:**
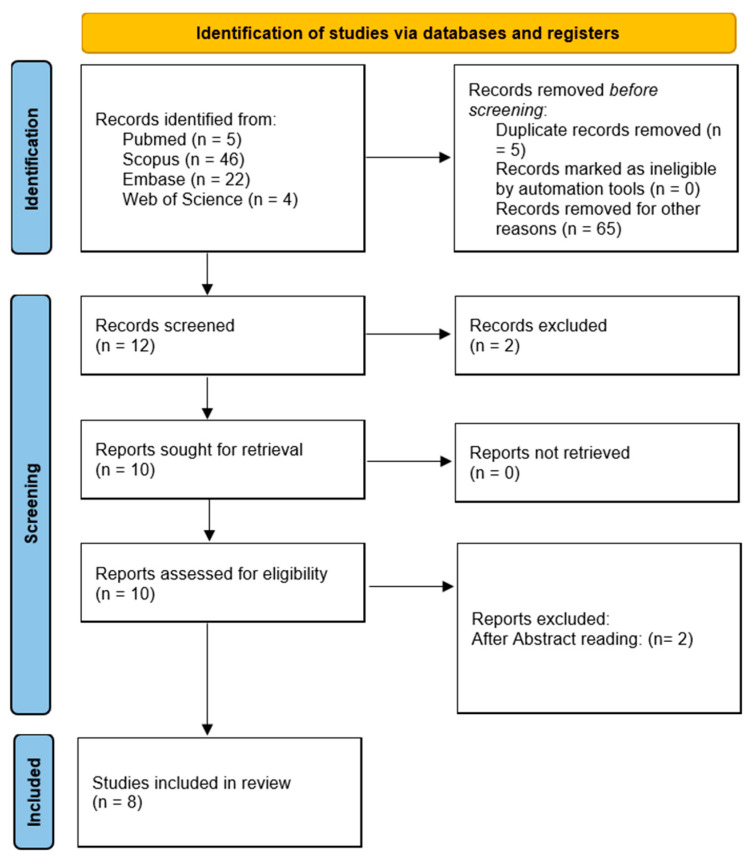
Flowchart article retrieval process.

A careful evaluation of searched studies was performed, and duplicates were removed, while titles and abstracts were screened by two reviewers independently in order to assess eligibility. Full-text articles were then retrieved and analyzed according to the predefined inclusion and exclusion criteria. The articles were all screened by two independent researchers Leopoldo Mauriello and Vitolante Pezzella, while in case of discrepancies a third reviewer (Alessandro Cuozzo) was involved when it was deemed necessary. The search and article selection process is summarized in Figure 1.

## 3. Relevant Sections

### 3.1. Oral Hygiene Ability and Motor Impairment

Motoneuron disease such as ALS in particular its bulbar onset and upper limb involvement may severely compromise patients’ ability to maintain proper oral hygiene level. ALS patients often record a progressive loss of function of their manual dexterity and tongue control which greatly increase the difficulties of both tooth brushing and plaque removal. Supporting infrastructure and caregiver assistance play therefore a crucial role in the reduction in oral disease risk. For instance, an online survey recorded that over 70% of patients with motoneuron disease felt they lacked assistance from their treatment teams regarding oral care, and those without support reported significantly poorer oral-health-related quality of life [12]. Similarly, clinical reports highlight the concept that caregivers must often perform daily hygiene activities as the disease progresses [13].

### 3.2. Caries and Periodontal Disease Prevalence

ALS patients generally record high rates of tooth decay and periodontal disease. In fact, oral motor dysfunction often reduces salivary flow contributing to both plaque accumulation and decay. However, a hospital-based cross-sectional study highlighted that while severe dental diseases were uncommon due to nursing care, maximum mouth opening was extremely restricted (mean 13.7 ± 7.4 mm) therefore making extremely difficult oral hygiene interventions [14]. Moreover, functional oral health metrics in ALS patients such as tongue coating and gingival inflammation seems to be linked to sialorrhea severity and upper limb deficits, underscoring higher caries and periodontal risks [15].

### 3.3. Salivary Dysfunction and Drooling

Salivary dysfunction likely manifests as sialorrhea which is a typical sign in many ALS patients particularly in bulbar-onset cases. Drooling derives from impaired muscle control rather than increased saliva production, and it is linked to greater tongue coating and gingival inflammation, increasing aspiration risk. A meta-analysis across 17 ALS cohorts found a sialorrhea prevalence of approximately 30.8%, with different degrees of severity ranging widely due to assessment method [16]. In clinical interventions, botulinum toxin injections into salivary glands seems to reduce drooling improving patient-reported quality of life in bulbar-onset ALS [17].

### 3.4. Impact of Oral Health on Quality of Life and Care

Oral health in ALS is linked to patient comfort, nutritional intake, social engagement, and overall well-being. An impaired oral status often due to tongue movement limitations, restricted mouth opening, and residual food debris can further increase dysphagia and therefore reduce quality of life. A case series used the Nordic Orofacial Test-Screening (NOT-S) observing a rapid decline in orofacial functions in bulbar ALS patients, yet personalized care managed to preserve oral comfort in all participants through follow-up period [15]. Additionally, cross-sectional research highlighted that insufficient oral hygiene is associated with worse oral-health-related quality of life (QoL) among MND (motoneuron disease) patients [12].

## 4. Results

Eight studies met the eligibility criteria and were included in the review. Oral health status in patients diagnosed with Amyotrophic Lateral Sclerosis (ALS) were investigated under several aspects. The evaluation was performed using various methodologies, assessment tools, and clinical settings. Across the included studies, patient cohorts were rather heterogeneous, with a mean age ranging from 52.8 to 70.7 years. The included studies analyzed different ALS subgroups, most commonly bulbar-onset versus spinal-onset phenotypes, and in some cases distinguished upper motoneuron (UMN)-predominant and lower motoneuron (LMN)-predominant forms. Main findings were synthesized in different paragraphs which considered relevant aspects to oral health in ALS, such as orofacial function, dental and periodontal status, swallowing and quality of life, and multidisciplinary care involvement. All findings are summarized in Table 1.

### 4.1. Orofacial Dysfunction and Functional Impairments

Multiple studies identified significant orofacial impairments in ALS patients, especially those with bulbar onset. Bergendal et al. (2017) [15] recorded greater deterioration in Nordic Orofacial Test-Screening (NOT-S) scores over time in patients with bulbar onset, compared to spinal-onset patients (mean NOT-S score from 5.6 to 6.1 vs. 0.7 to 3.2, respectively). Similarly, Punet et al. (2017) [18] observed that bulbar-involved ALS patients showed a severe functional jaw limitations in chewing, swallowing, and speaking, conversely to those with non-bulbar ALS and controls.

Punet et al. (2018) [19] further confirmed these findings, showing that bulbar-onset ALS patients had the most significant reductions in both mouth opening and bite force, with 30% experiencing traumatic oral self-injury and 10% requiring treatment. Nakayama et al. (2021) [14] also documented severe restriction in mouth opening (mean: 13.7 ± 7.4 mm) and a negative correlation with disease duration and tracheostomy-positive pressure ventilation (TPPV) use.

### 4.2. Oral Health Status and Disease Characteristics

Different studies assessed oral hygiene, periodontal status, and dental conditions in ALS populations. De Sire et al. (2020) [20] found that all ALS patients presented poor oral health indicators, including gingival inflammation, tongue coating, and food debris independently from their functional status. Significant correlations were observed between sialorrhea and poor BOHSE (Brief Oral Health Status Examination) and WTCI (Winkel Tongue Coating Index) scores (*p* = 0.01, *p* = 0.04), and reduced tongue mobility was evidenced mainly in bulbar-onset patients.

In contrast, Tay et al. (2013) [21] observed that most ALS patients in their Australian cohort maintained relatively good oral health. Despite of the fact that 10 out of 27 dentate participants requiring restorative treatments or extractions, no participants showed periodontal probing depths over 3 mm.

Nakayama et al. (2021) [14] highlighted that hospitalized ALS patients receiving regular nursing oral care showed no significant differences in intraoral clinical features such as calculus, tongue anomalies, or saliva flow across different disease stages, focusing on the potential effectiveness of consistent care.

### 4.3. Swallowing Function and Quality of Life

Swallowing disorders were analyzed by Franceschini et al. (2015) [22] and Paris et al. (2012) [23]. Both studies showed that approximately 70% of spinal-onset ALS recorded both dysarthria and dysphagia, the most severe cases, which were related to reduced scores on the SWAL-QOL (Swallowing Quality of Life), especially when it comes to communication and eating fear. Paris et al. confirmed that oropharyngeal dysphagia significantly worsened quality of life, affecting both social and mental health aspects (*p* < 0.001). Patients with bulbar involvement recorded more severe impairments on the ALSFRS (Amyotrophic Lateral Sclerosis Functional Rating Scale), SWAL-QOL, and dysphagia severity scales.

**Table 1:** Characteristics and clinical findings of the analyzed studies.

Authors	Study Design	Setting	N° of Patients	Gender (Male/ Female)	Mean Age (Years)	Oral General Reported Conditions	Type of Amyotrophic Lateral Sclerosis	Assessment Tools	Results (Main Outcome of the Analyzed Study)	Conclusion
Paris et al. (2012) [23]	Cross-sectional observational study	Neuromuscular Clinic, Rouen University Hospital, France	30	18 M/12 F	62 ± 13	ALS diagnosis confirmed by El Escorial criteria; varied symptoms including bulbar involvement.	ALS diagnosis with bulbar and spinal involvement.	Videofluoroscopic barium swallow Dysphagia outcome severity scale ALS Functional Rating Scale (ALSFRS) Norris scores for cranial nerve involvement SWAL-QoL questionnaire for quality of life Amyotrophic Lateral Sclerosis (ALS) clinical assessments	Oropharyngeal dysphagia negatively impacts quality of life in ALS patients, especially affecting social interactions, mental health, and causing emotional burden. Quality of life was significantly worse in dysphagic patients, notably in aspects such as eating burden, duration, desire, fear, communication, mental health, and social life, with statistical significance (*p* < 0.001 to *p* < 0.05)	Oropharyngeal dysphagia is a common and impactful symptom in ALS, significantly altering patients’ quality of life, particularly social and mental health aspects. Identifying and managing swallowing difficulties are crucial in improving overall well-being.
Tay et al. (2013) [21]	Cross-sectional clinical study	Multidisciplinary MND clinic at Bethlehem Hospital, Melbourne, Victoria, Australia	33	N.A.	N.A.	8 out of 33 reported regular dental visits 10 of 27 dentate participants required extractions/restorations due to retained roots or caries 3 participants had non-carious cavities, lost restorations, or fractured cusps No participant exhibited probing depths of more than 3 mm	Motoneuron disease	Clinical examination including charting of dentition, restorations, caries, periodontal status Plaque and Gingival Indices Oral Health Assessment Tool (OHAT)	Oral health status was found to be generally good and not significantly affected by advanced MND, possibly due to the motivation and involvement of both participants and healthcare teams.	Oral health in patients with advanced MND was maintained at a good level, and the motivation of both patients and clinicians likely contributed. The involvement of dental professionals as part of the multidisciplinary care team is recommended.
Franceschini et al. (2015) [22]	Observational cross-sectional study	Dysphagia Clinic, UNICAMP Hospital, Brazil	17	11 M/ 6 F	52.8 ± 10.9	Dysarthria, dysphagia, reduced QOL related to swallowing	Spinal-onset only	SWAL-QOL Speech Subscale of ALS Severity Scale FEES, Dysphagia Severity Scale FOIS	- 70% had both dysarthria and dysphagia. - Severity of both conditions correlated with lower SWAL-QOL scores, particularly in communication, fear of eating, and symptom frequency domains.	Dysarthria and dysphagia commonly occur in spinal-onset ALS and significantly reduce
Punet. et al. (2017) [19]	Cross-sectional observational study	Motor Neuron Disease Unit, Bellvitge University Hospital, Barcelona, Spain;	153 patients with ALS 23 healthy Patients	54% M/ 46% F (ALS group) 44% M/ 56% F (healthy group)	62 (ALS group) 51 (healthy group)	Functional jaw limitations; some received botulinum toxin or non-invasive ventilation	Bulbar involvement and non-bulbar ALS	Jaw Functional Limitation Scale-8 items (JFLS-8) Functional Rating Scale (ALSFRS)	- Non-bulbar ALS had similar jaw function to controls. - Bulbar-involved ALS had significant chewing, swallowing, and talking difficulties. - Worst performance seen in balanced UMN + LMN group.	Bulbar ALS is associated with functional limitation in mastication; balanced UMN/LMN involvement leads to the worst impairments.
Bergendal et al. (2017) [15]	Observational longitudinal case series (quality improvement project)	National Oral Disability Centre for Rare Disorders, Jönköping, Sweden	14	5 M/9 F	62.8	All dentate patients, mean number of teeth: 26.7; good baseline oral health	8 bulbar-onset 6 spinal-onset	Nordic Orofacial Test-Screening (NOT-S) Dental examination Panoramic radiography	Bulbar group showed worse NOT-S scores (initial 5.6 → final 6.1) vs. spinal group (0.7 → 3.2) - Most patients required oral hygiene assistance in advanced stages - No further dental treatment needed after baseline - Oral health remained stable	Orofacial functions progressively declined, especially in bulbar cases. With regular support and hygiene aids, oral comfort and health were maintained throughout ALS progression.
Punet et al. (2018) [18]	Cross-sectional controlled study	ALS Unit, Bellvitge University Hospital, Spain	153 ALS patients, 23 controls	54% M/ 46% F (ALS group) 44% M/ 56% F (healthy group)	64.2 (ALS group) 52 (healthy group)	Reduced mouth opening, bite force; traumatic self-injury (tongue, cheek, lip); increased sialorrhea	Bulbar, spinal, and respiratory onset	DC/TMD protocol mandibular movement measures bite force finger-thumb grip force tests	Maximum unassisted mouth opening was significantly reduced in ALS patients (both spinal- and bulbar-onset) compared to controls (*p* < 0.001) Maximum mouth protrusion was significantly reduced, with (*p* < 0.001) Finger–thumb grip force was significantly lower, with (*p* < 0.001) Bite force was significantly decreased in bulbar-onset patients (*p* < 0.001) but not explicitly in spinal-onset patients The prevalence of traumatic mucosal injuries was higher in ALS patients compared to controls (29.9% vs. 8.7%, *p* = 0.03) No significant increase in TMD prevalence was noted (*p* > 0.05)	ALS patients, particularly with bulbar onset, show significant reduction in masticatory function and increased self-injury. Dentists should be included in the care team.
Nakayama al. (2018) [14]	Single-center, cross-sectional observational study	Sayama Neurology Hospital Japan	50	31 M / 19 F	70.7	The maximum mouth opening was significantly restricted, with an average of 13.7 ± 7.4 mm. Oral health parameters such as dental calculus, bleeding on probing, tongue atrophy or hypertrophy, and tongue coating were assessed, with no significant difference related to disease or TPPV duration	N.A.	Oral health assessments included the community periodontal index (CPI), maximum mouth opening, saliva flow rate, tongue anomalies, and dental conditions	Severe dental disease was uncommon among the hospitalized ALS patients receiving nursing oral care Mouth opening was markedly restricted and negatively correlated with disease duration and TPPV duration No significant correlations were observed between other intraoral parameters and disease or TPPV duration	Early intervention targeting mouth opening restrictions is essential, and maintaining oral health in hospitalized ALS patients can be effectively managed by nurses despite the challenge of restricted mouth opening
De Sire et al. (2021) [20]	Cross-sectional clinical study	ALS Center, Neurology Unit, University Hospital “Maggiore della Carità”, Novara, Italy	37	12 M/25 F	61.19 ± 11.56	Poor oral health across all patients (e.g., food debris, tongue coating, gingival inflammation) Strong correlation with sialorrhea and reduced tongue mobility, especially in bulbar-onset ALS	Spinal-onset (SO) Bulbar-onset (BO)	ALS Functional Rating Scale-Revised (ALSFRS-R) Brief Oral Health Status Examination (BOHSE) Winkel Tongue Coating Index (WTCI) Oral Food Debris Index (OFDI) Gingival Index (GI) Oral Hygiene Index (OHI) New Method of Plaque Scoring (NMPS)	Oral health was impaired in all patients, independent of functional status Sialorrhea significantly correlated with poor BOHSE and WTCI scores (*p* = 0.01, *p* = 0.04) BO patients had significantly lower tongue mobility OFDI negatively correlated with upper limb function (ALSFRS-R UL, *p* = 0.03) BOHSE and WTCI were significantly negatively correlated with survival time Oral health indices not correlated with cognitive impairment	A poor oral health status is common in ALS patients and may contribute to reduced functional performance and survival time. The study highlights the need for integrating oral healthcare and rehabilitation into the multidisciplinary management of ALS. Oral screening and targeted intervention should become a regular part of ALS care

**Table abbreviations: N.A**.: not available; **ALS**: amyotrophic lateral sclerosis; **NOT-S**: Nordic Orofacial Test-Screening; **UMN**: upper motoneuron; **LMN**: lower motoneuron; **JFLS**: Jaw Functional Limitation Scale; **ALSFRS**: Amyotrophic Lateral Sclerosis Functional Rating Scale; **SWAL-QOL**: Swallowing Quality of Life; **FEES**: Fiberoptic Endoscopic Evaluation of Swallowing; **FOIS**: Functional Oral Intake Scale; **DC/TMD**: Diagnostic Criteria For Temporomandibular Disorders; **OHAT**: Oral Health Assessment Tool; **ALSFRS-R**: ALS Functional Rating Scale-Revised; **BOHSE**: Brief Oral Health Status Examination; **WTCI**: Winkel Tongue Coating Index; **OFDI**: Oral Food Debris Index; **GI**: Gingival Index; **OHI**: Oral Hygiene Index; **NMPS**: New Method of Plaque Scoring; **CPI**: Community Periodontal Index.

### 4.4. Role of Supportive Care in Maintaining Oral Health

A multidisciplinary approach together with nursing care is needed in patients with ALS in order to preserve oral health. Bergendal et al. (2017) [15] showed that a regular oral hygiene may be able to maintain oral comfort even if a pathology worsening is recorded through the different phases of illness progression, even when orofacial functional decline is evident. Both Tay et al. (2013) and Nakayama et al. (2021) [14,21] recorded that even when a restricted mouth opening is present, proper oral health may be maintained through the help of caregivers and dentists’ focused intervention.

## 5. Discussion

The main objective of this scoping review was to examine oral health status of patients affected by amyotrophic lateral sclerosis (ALS), focusing on the possibility to maintain oral hygiene evaluating the prevalence of common dental and periodontal pathologies and if the diagnosis and treatment of these conditions may positively affect their quality of life. ALS is a complex neurodegenerative disorder or rather a syndrome [24] that progressively compromises several neuromuscular functions, often leading to limitation in performing essential daily activities and among them oral hygiene maintenance. The data analyzed were highly variable since a considerable quantity of conditions and disease manifestations were recorded. However, it seems that patients with bulbar-onset ALS tend to show the most severe orofacial impairments, in particular: reduced tongue mobility, jaw function limitations, bite force reduction, increased sialorrhea, and traumatic self-injuries to the oral mucosa. Bergendal et al. and Punet et al. [15,18] demonstrated significantly worse NOT-S and JFLS-8 scores among bulbar-onset ALS patients compared to spinal-onset or healthy controls; therefore, they recorded a progressive deterioration of orofacial function. De Sire et al. [20] confirmed the high prevalence of poor oral status highlighting a significant association between sialorrhea, tongue mobility, and oral hygiene indices like the BOHSE and WTCI. These deficits were also linked to shorter survival time. Franceschini et al. and Paris et al. focused on the link between dysphagia, dysarthria, and reduced swallowing-related quality of life, showing a significant impact on communication, eating, and mental health domains [22,23]. This strongly supports the hypothesis that oral dysfunction in ALS is not only physical but also psychosocial, affecting patients’ interaction with their environment and their emotional well-being. This is in line with a recent scoping review showing that education-related strategies, behavior therapy, counseling, and social and psychotherapy interventions may improve both patient’s quality of life and their family [25]. The results of the review seem to be also in line with several studies that demonstrate a strong association between bulbar dysfunction and oral deterioration, with drooling, poor salivary clearance, and plaque accumulation being common features [26]. A meta-analysis by Giess et al. also reported sialorrhea prevalence above 30% in ALS, and interventions like botulinum toxin therapy seems to reduce discomfort and improve quality of life [27]. On the contrary, Tay et al. and Nakayama et al. suggested that institutionalized patients with consistent dental and nursing care could maintain adequate oral health, even in advanced disease stages. These contrasting results highlight the crucial role of caregivers and the importance of structured oral health protocols within multidisciplinary ALS management. In this context, the preservation of oral health becomes a clinical necessity. Poor oral health in ALS is linked not only to pain and discomfort, but also to systemic complications like aspiration pneumonia, which is one of the leading causes of death in ALS patients [28]. Furthermore, the treatment of oral conditions, such as periodontitis, may also be useful in the systemic treatment of ALS; in fact, some periodontal pathogens and chronic oral inflammation may increase systemic inflammatory burden [29,30], which may lead to a faster disease progression. Thus, the role of good oral hygiene in preventing aspiration and secondary infections cannot be underestimated, especially in patients with compromised swallowing and coughing reflexes. In addition, poor oral status may contribute to reduced nutritional intake due to pain or fear of choking. In a clinical study, it was also evaluated that ALS patients may require an increase in terms of energy. This underline that a well-balanced diet may be necessary to avoid disease progression [31]. From a quality-of-life perspective, oral impairments may severely limit social participation, speech, smiling, and basic expressions of emotion. Oral health, therefore, is a determinant of psychosocial well-being in ALS patients.

Considering all these factors dentists may play an important role within the multidisciplinary care team. A dental expert may help both in monitoring and managing ALS-related oral complications, ranging from temporomandibular disorders and reduced mouth opening to gingivitis, caries, and oral injuries. Dentists may also be able to provide tailored minimally invasive preventive procedures and therapeutic care and could also educate caregivers on customized oral hygiene protocols. Moreover, dental involvement can aid in the early detection of dysphagia, oral infections, or mucosal lesions. In particular, the treatment of some oral pathologies such as periodontitis may also benefit from adjunctive therapies such as the use of molecules with antibacterial and antioxidant properties, which may be useful not only for improving clinical outcomes, but also for systemic treatment [32,33]. The results of the following review highlight several preventive strategies that can be considered to improve oral health in ALS affected patients, including the use of appropriate oral hygiene devices (e.g., electric toothbrushes, interdental devices, dental floss, high-fluoride toothpaste, topical varnishes and remineralizing agents) in order to reduce the risk of caries. Xerostomia may be treated with artificial saliva substitutes, gels and proper hydration; conversely, sialorrhea may find benefit with the use of suction devices, pharmacological intervention or even botulinum toxin under medical supervision. Regarding nutrition dentists may help dietitians to promote an appropriate diet plan. Improving oral health and reducing bacterial load may lead to lower risk of aspiration pneumonia, a common risk in ALS patients. Finally, in advanced disease stages, dentists should focus on a palliative approach, managing pain and preserving oral function for as long as possible. These practical recommendations reflect the clinical relevance of the study reinforcing the need for preventive and patient-centered strategies trying to focus on the central role of dental professionals within the multidisciplinary care of ALS patients. The role of the dentist and their active participation in multidisciplinary planning can ensure that oral health remains integrated with nutritional, respiratory, and communicative functions. Despite these insights, there are several limitations to the present study that must be acknowledged. Most studies were observational and cross-sectional, with relatively small sample sizes. Assessment tools were widely variable, and standardized criteria for evaluating oral status in ALS are lacking. Furthermore, the disease heterogeneity (bulbar vs. spinal onset, institutionalized vs. community living) adds a further confounding factor making it difficult to generalize the data obtained. Future research should focus on randomized trials to evaluate the efficacy of preventive oral care strategies. In addition, developing ALS-specific oral health guidelines and training protocols for caregivers and clinicians may also be useful.

## 6. Conclusions

ALS patients are subject to progressive neuromuscular conditions, often leading to a progressive weakness, fatigue and finally respiratory distress. Several oral conditions may affect patients’ quality of life and social interaction. Improving and preserving a proper oral health status may be useful to reduce oral deterioration. Thus, despite the limitations of the present study, such as, the heterogeneity of the analyzed studies, the lack of a unique evaluation criteria and the small number of included studies, it seems reasonable to conclude that dentists and oral hygienists should play an important role in multidisciplinary ALS care teams to diagnose and treat oral health conditions, from temporomandibular disorders to caries and periodontitis.

## Data Availability

All data are available and published online.

## Abbreviations

The following abbreviations are used in this manuscript:

ALS	Amyotrophic lateral sclerosis
NOT-S	Nordic Orofacial Test-Screening
UMN	Upper motoneuron
LMN	Lower motoneuron
JFLS	Jaw Functional Limitation Scale
ALSFRS	Amyotrophic Lateral Sclerosis Functional Rating Scale
SWAL-QOL	Swallowing Quality of Life
FEES	Fiberoptic Endoscopic Evaluation of Swallowing
FOIS	Functional Oral Intake Scale
DC/TMD	Diagnostic Criteria For Temporomandibular Disorders
OHAT	Oral Health Assessment Tool
ALSFRS-R	ALS Functional Rating Scale-Revised
BOHSE	Brief Oral Health Status Examination
WTCI	Winkel Tongue Coating Index
OFDI	Oral Food Debris Index
GI	Gingival Index
OHI	Oral Hygiene Index
NMPS	New Method of Plaque Scoring
CPI	Community Periodontal Index

## Appendix A. Search Strategies

Database	Search Strategy	Filters/Limitations
PubMed	(“amyotrophic lateral sclerosis” [MeSH Terms] OR (“amyotrophic” [All Fields] AND “lateral” [All Fields] AND “sclerosis” [All Fields]) OR “amyotrophic lateral sclerosis” [All Fields]) AND ((“oral health” [MeSH Terms] OR (“oral” [All Fields] AND “health” [All Fields]) OR “oral health” [All Fields]) AND “status” [All Fields]) AND ((casereports [Filter] OR clinicalstudy [Filter] OR clinicaltrial [Filter] OR controlledclinicaltrial [Filter] OR randomizedcontrolledtrial [Filter]) AND (humans [Filter]) AND (English [Filter]))	Humans, English, case reports, clinical study, clinical trial, controlled clinical trial, randomized controlled trial
Scopus	(TITLE-ABS-KEY (“amyotrophic lateral sclerosis”) OR TITLE-ABS-KEY (ALS)) AND (TITLE-ABS-KEY (“oral health status”) OR (TITLE-ABS-KEY (“oral health”) AND TITLE-ABS-KEY (status)))	Document type: article; language: English
Embase	(‘amyotrophic lateral sclerosis’/exp OR ‘amyotrophic lateral sclerosis’) AND (‘oral health status’ OR (‘oral health’/exp AND status)) AND ([english]/lim AND [humans]/lim) AND ([article]/lim OR [clinical study]/lim OR [clinical trial]/lim OR [randomized controlled trial]/lim OR [case report]/lim)	Humans, English, case report, clinical study, clinical trial, randomized controlled trial
Web of Science	TS = (“amyotrophic lateral sclerosis” OR ALS) AND TS = (“oral health status” OR (“oral health” AND status))	Language: English; document type: article
